# Effect of Delgocitinib Cream on Health‐Related Quality of Life in Patients With Moderate to Severe Chronic Hand Eczema

**DOI:** 10.1111/cod.70114

**Published:** 2026-03-03

**Authors:** Andrea Bauer, Lyn Guenther, Richard Woolf, Eydna Didriksen Apol, Douglas Maslin, Nanna Nyholm, Henrik Thoning, Marie Louise Schuttelaar

**Affiliations:** ^1^ Department of Dermatology, University Allergy Centre, University Hospital Carl Gustav Carus Technical University Dresden Germany; ^2^ Division of Dermatology, Department of Medicine Western University London Ontario Canada; ^3^ St John's Institute of Dermatology Guy's and St Thomas' NHS Foundation Trust London UK; ^4^ LEO Pharma A/S Ballerup Denmark; ^5^ Department of Dermatology, University Medical Center Groningen University of Groningen Groningen the Netherlands

**Keywords:** contact dermatitis, eczema, hand eczema impact scale, health‐related quality of life

## Abstract

**Background:**

Chronic Hand Eczema (CHE) is a multifactorial, fluctuating inflammatory disease of the hands and wrists that negatively impacts patients' health‐related quality of life (HRQoL).

**Objectives:**

To assess the effects of delgocitinib cream versus cream vehicle on HRQoL.

**Methods:**

DELTA 1 and DELTA 2 were phase 3 trials of identical design in which adults with moderate to severe CHE were randomised (2:1) to double‐blind treatment with delgocitinib cream 20 mg/g or cream vehicle twice daily for 16 weeks. Data were pooled from both trials and changes in EQ‐5D, Dermatology Life Quality Index (DLQI), and Hand Eczema Impact Scale (HEIS), including the HEIS Proximal Daily Activity Limitations (PDAL) and HEIS Embarrassment (Emb) subsets, were compared.

**Results:**

Across the two trials, 639 patients were randomised to delgocitinib cream and 321 to cream vehicle. Delgocitinib cream resulted in significantly greater improvement in EQ‐5D, DLQI, HEIS, HEIS PDAL, and HEIS Emb compared with cream vehicle at week 16 (all *p* < 0.001). More patients achieved clinically meaningful improvement from baseline in patient‐reported outcomes at week 16 (all *p* < 0.001), and the mean time spent in an improved HRQoL state increased with delgocitinib cream.

**Conclusions:**

Delgocitinib cream resulted in clinically significant improvement in HRQoL compared with cream vehicle.

**Clinical Trial ID:**

DELTA 1 (NCT04871711) and DELTA 2 (NCT04872101).

## Graphical abstract

Patient‐reported outcomes are an important complement to physician‐assessed clinical outcome measures in Chronic Hand Eczema. In a pooled analysis of two randomised, controlled trials, twice‐daily treatment with delgocitinib cream 20 mg/g significantly improved the health‐related quality of life of patients with moderate to severe Chronic Hand Eczema compared with placebo.

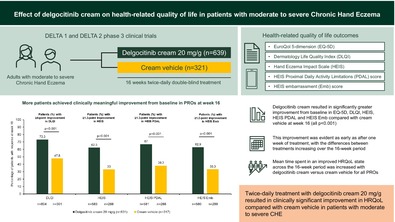

## 1 Introduction

Chronic Hand Eczema (CHE) is a multifactorial, fluctuating chronic inflammatory disease of the hands and wrists. Patients may experience itch and pain and various signs including erythema, vesicles, edema, scaling, lichenification, hyperkeratosis, and fissures [1, 2, 3]. Given how central hands are to daily living, CHE can have a profound negative impact on patients' quality of life, physical functioning and ability to work [4, 5, 6]. Since the hands are visible, skin eruptions can result in a considerable psychological burden, including self‐consciousness and embarrassment that affects social life, leisure activities, and employment [7, 8]. Patients with hand eczema also have significantly higher levels of anxiety and depression compared to healthy controls [9, 10]. Itch and pain can result in impaired sleep and reduced ability to perform tasks such as holding or gripping objects, wet‐work, domestic chores, leisure activities, self‐care, and caring for others [5, 6, 7, 8, 11]. The ability to work may be impacted with patients experiencing presenteeism, absenteeism and/or job loss [12, 13]. The negative impact of moderate to severe CHE on quality of life has been reported to be comparable to that of psoriasis, atopic dermatitis and chronic non‐dermatological diseases, including vision disorders, hepatitis, and some types of cancer [14, 15].

Patients with moderate to severe CHE have limited treatment options. High‐potency topical corticosteroids (TCS) are often used, but use, particularly if prolonged, may be associated with skin atrophy, contact allergy, impaired barrier function, reduced hand dexterity, and worsening of CHE signs and/or symptoms [16, 17, 18]. Alitretinoin, a systemic retinoid, is approved in some countries for severe CHE unresponsive to TCS. However, its use requires medical and laboratory monitoring and a pregnancy prevention programme for women of child‐bearing potential [19].

Delgocitinib cream is a first‐in‐class topical pan‐Janus kinase (JAK) inhibitor that prevents the activation of JAK–STAT‐mediated cytokine signalling pathways [20]. Treatment with delgocitinib cream resulted in significant improvements in health‐related quality‐of‐life in a 16‐week dose‐ranging phase 2b trial in patients with moderate to severe CHE [21].

In two pivotal randomised, double‐blind, multicentre phase 3 trials (DELTA 1 and DELTA 2), twice‐daily application of delgocitinib cream 20 mg/g resulted in superior efficacy versus cream vehicle and was well‐tolerated over 16 weeks [22]. Here, we report the effects of delgocitinib cream 20 mg/g versus cream vehicle on HRQoL in data pooled from the DELTA 1 and DELTA 2 trials.

## 2 Methods

DELTA 1 (NCT04871711) and DELTA 2 (NCT04872101) were phase 3 trials of identical design. In both trials, adults with moderate to severe CHE according to a modified Investigator's Global Assessment for Chronic Hand Eczema (IGA‐CHE) score of 3 (moderate) or 4 (severe) were randomised (2:1) to double‐blind treatment with delgocitinib cream 20 mg/g or cream vehicle twice daily for 16 weeks. Patients were required to have a recent history of inadequate response to TCS, or TCS were documented to be medically inadvisable. Both trials were conducted in accordance with the Declaration of Helsinki, Good Clinical Practice guidelines, and applicable regulatory requirements, and were approved by the local institutional review board or independent ethics committee of each institution. All patients provided written informed consent before undergoing any study‐related procedures, including consent to publish anonymised photographs. The methodology of these trials has previously been reported [22].

HRQoL was evaluated using the EQ‐5D‐5L questionnaire score and visual analogue scale (EQ VAS), Dermatology Life Quality Index (DLQI), Hand Eczema Impact Scale (HEIS), HEIS Proximal Daily Activity Limitations (PDAL) score and the HEIS embarrassment (Emb) score. The EQ‐5D is a generic preference‐based measure that describes health states on the day completed through the generation of a utility score based on levels of impairment on five dimensions (mobility, self‐care, usual activities, pain/discomfort, and anxiety/depression); utility scores represent health states anchored at perfect health (1) and death (0) [23]. The EQ‐5D‐5L index score is derived from the 5 dimensions and was converted from the 5 L system to the 3 L system using the EQ‐5D‐5L crosswalk value set. The index score ranges from −0.594 to 1.0 (based on the UK country‐specific value set), with a higher score indicating a better health status. EQ VAS captures overall assessment of health on a scale from 0 (worst health imaginable) to 100 (best health imaginable). The DLQI is a dermatology‐specific questionnaire that assesses the impact of skin disease over the previous 7 days on symptoms, feelings, daily activities, leisure, work and school, personal relationships and the impact of treatment. DLQI scores range from 0 to 30, with 0–1 no effect at all on patient's life, 2–5 a small effect, 6–10 a moderate effect, 11–20 a very large effect, and 21–30 an extremely large effect [24]. A decrease in DLQI represents an improvement. The minimum clinically important difference (MCID), that is the smallest within‐individual change from baseline that is perceived as meaningful by the patient, for the DLQI is considered to be 4 [25]. The HEIS was specifically developed to assess the impact of CHE, with the total HEIS score the average of nine items assessed over the previous 7 days: ability to use soaps/cleaning products, difficulty with housework, difficulty with washing, embarrassment with appearance of hands, dislike with the appearance of hands, frustration, quality of sleep, difficulty with work, and difficulty holding/gripping objects [26]. Each item is scored on a 5‐point scale ranging from 0 (no impact at all) to 4 (extreme impact). The HEIS PDAL and HEIS Emb scores are multi‐item domain subsets of the full HEIS; HEIS PDAL is scored by taking an average of three items (ability to use soaps/cleaning products, difficulty with housework, and difficulty with washing) and HEIS Emb is scored by taking an average of two items (embarrassment over hands due to eczema and dislike of appearance of hands). An appropriate threshold for defining a within‐individual improvement is a reduction of 1.3 points for the HEIS and HEIS PDAL scores and a reduction of 1.5 points for the HEIS Emb score [26]. Scores are summarised in Table S1.

## 3 Endpoints and Statistical Analysis

All analyses used the primary estimand (composite estimand). For categorical endpoints, the composite estimand strategy for intercurrent events considered data collected after initiation of rescue treatment or after permanent discontinuation of trial treatment as non‐response. Any other missing data for an endpoint of interest were also imputed as non‐response. For continuous endpoints, worst observation carried forward (including baseline value) was applied as non‐response. Efficacy was assessed based on the full analysis set, which consisted of all randomised patients exposed to trial treatment. Patients without baseline patient‐reported outcome (PRO) values were excluded.

HRQoL was evaluated by determining changes from baseline to week 16 for EQ‐5D, DLQI, HEIS, HEIS PDAL, and HEIS Emb. Changes from baseline were analysed using an ANCOVA model with study ID, treatment, region, baseline IGA‐CHE score and baseline PRO value as independent variables. Least squares (LS) means were estimated, and treatment effects were evaluated by *t*‐tests. The proportions of patients achieving a clinically meaningful improvement were also assessed, using previously reported thresholds for each score. Treatment differences in the proportions of patients achieving the endpoint of MCID from baseline were assessed using the Cochran–Mantel–Haenszel risk difference, stratified by study ID, region, and baseline IGA‐CHE score. As the EQ‐5D is primarily a tool for health economic analyses, there is no consensus around the concept of MCID and thus no generally accepted threshold.

Due to the fluctuating nature of CHE, estimated cumulative incidence of patients achieving a clinically meaningful improvement was also assessed (i.e., the proportion of patients to have achieved a response at any timepoint up to and including the timepoint being assessed) for each measure (other than EQ‐5D, due to lack of an accepted MCID). Adjusted mean time spent in response (area under the curve) was also assessed in all patients and in the subgroup of patients who achieved a response. Cumulative incidence was estimated using the Aalen‐Johansen estimator with permanent discontinuation of trial treatment and initiation of rescue treatment as competing risks.

## 4 Results

Across the two trials, 639 patients were randomised to delgocitinib cream 20 mg/g and 321 to cream vehicle; one patient randomised to delgocitinib did not receive any study treatment so was excluded from the full analysis set. A further seven patients in the delgocitinib group and four patients in the cream vehicle group did not have baseline EQ‐5D, DLQI, or HEIS values so were excluded. Most patients were white (90%), female (64%), with moderate CHE disease severity (72%) at baseline and a mean duration of CHE of approximately 10 years. Mean EQ‐5D, DLQI, and HEIS scores at baseline were similar between groups. Baseline characteristics are summarised in Table 1.

**TABLE 1:** Baseline characteristics.

	Delgocitinib cream 20 mg/g (*N* = 639)	Cream vehicle (*N* = 321)
Age, mean (SD), years	44.8 (14.5)	42.7 (14.2)
Sex, female, *n* (%)	406 (63.5%)	212 (66.0%)
Race, *n* (%)		
White	578 (90.5%)	290 (90.3%)
Black or African American	5 (0.8%)	2 (0.6%)
Asian	22 (3.4%)	12 (3.7%)
Other^a^ or not reported	34 (5.3%)	17 (5.3%)
Duration of CHE, years, mean (SD)	9.6 (11.0)	10.0 (11.2)
Age at onset of CHE, years, mean (SD)	35.2 (17.0)	32.8 (16.8)
Presence of atopy, *n* (%)	244 (38.2%)	128 (39.9%)
IGA‐CHE score, *n* (%)		
Moderate	457 (71.5%)	230 (71.7%)
Severe	182 (28.5%)	91 (28.3%)
HECSI score, mean (SD)	71.1 (43.0)	72.5 (47.3)
EQ‐5D‐5L index score, mean (SD)	0.6 (0.2)	0.6 (0.2)
DLQI score, mean (SD)	12.4 (6.1)	12.6 (6.7)
DLQI score, category^b^		
< 4	27 (4.3%)	16 (5.0%)
≥ 4	604 (95.7%)	301 (95.0%)
HEIS score, mean (SD)	2.5 (0.8)	2.5 (0.9)
HEIS PDAL score, mean (SD)	2.6 (0.9)	2.6 (0.9)
HEIS Emb score, mean (SD)	2.8 (1.0)	2.8 (1.0)

^a^ Native Hawaiian or other Pacific Islander, American Indian or Alaska Native, multiple or other.

^b^ Change in DLQI score of ≥ 4 considered clinically important; patients with baseline < 4 excluded from analysis of % of patients achieving ≥ 4 reduction.

### 4.1 EQ‐5D

**FIGURE 1:**
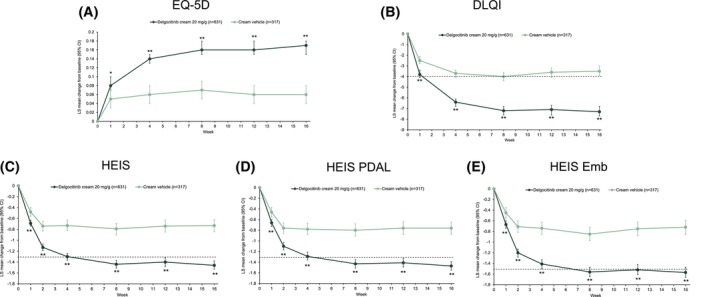
Changes from baseline to week 16 in (A) EQ‐5D, (B) DLQI, (C) HEIS, (D) HEIS PDAL, and (E) HEIS Emb in patients treated with delgocitinib cream or cream vehicle. *p < 0.05; **p ≤ 0.001. Dotted line represents the minimum clinically important difference from baseline (EQ‐5D, not applicable). DLQI, Dermatology Life Quality Index; Emb, Embarrassment; EQ‐5D, EuroQol 5‐dimension HEIS; Hand Eczema Impact Scale; PDAL, Proximal Daily Activity Limitations.

**FIGURE 2:**
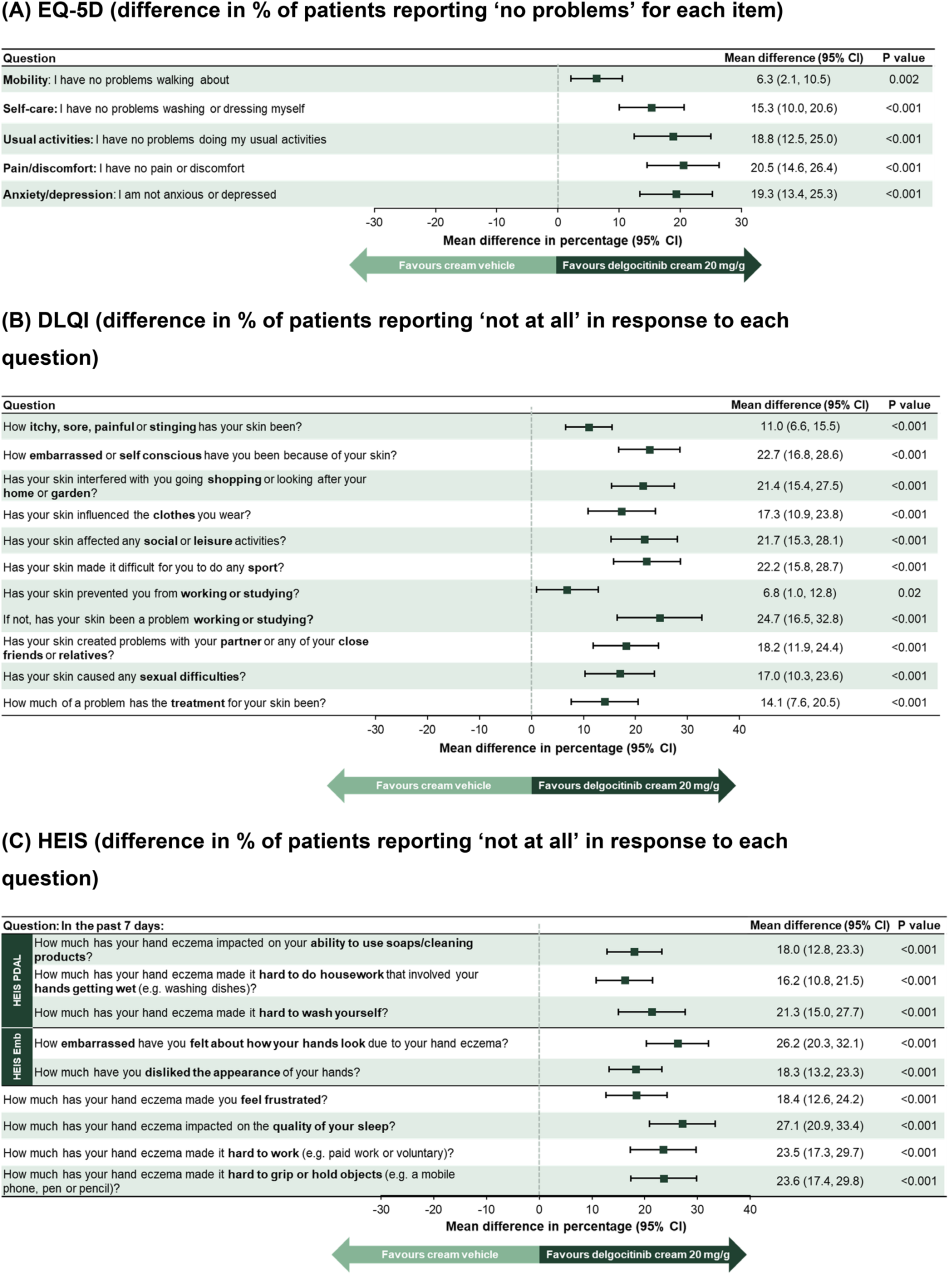
Estimated treatment differences in individual items/domains of EQ‐5D, DLQI and HEIS (including HEIS PDAL and HEIS Emb). (A) EQ‐5D (difference in % of patients reporting ‘no problems’ for each item). (B) DLQI (difference in % of patients reporting ‘not at all’ in response to each question). (C) HEIS (difference in % of patients reporting ‘not at all’ in response to each question).

Treatment with delgocitinib cream resulted in a significantly greater improvement in EQ‐5D from baseline compared to cream vehicle. The difference between treatments in favour of delgocitinib cream was significant (*p* = 0.022) as early as week 1 and continued to increase up to week 16, at which timepoint the LS mean change [SE] from baseline was 0.17 ± 0.01 with delgocitinib cream versus 0.06 ± 0.01 with cream vehicle (treatment difference [95% CI] 0.11 [0.08, 0.13], *p* < 0.001) (Figure 1A). Mean improvements were significantly greater with delgocitinib cream versus cream vehicle across all five EQ‐5D dimensions at week 16 (Figure 2 and Table S2).

### 4.2 Dermatology Life Quality Index

Delgocitinib cream resulted in significantly greater improvement (i.e., reduction) in DLQI from baseline compared to cream vehicle, from week 1 onwards. Treatment difference increased until week 16, when LS mean change [SE] was −7.3 ± 0.2 with delgocitinib cream versus −3.5 ± 0.3 with cream vehicle (treatment difference −3.8, [95% CI: −4.5, −3.0], *p* < 0.001) (Figure 1B). Improvement in DLQI at week 16 was significantly greater with delgocitinib cream versus cream vehicle across all 10 DLQI items, in particular those concerning effects on work, social, leisure, and other daily activities, and skin‐related embarrassment or self‐consciousness (Figure 2 and Table S2).

**FIGURE 3:**
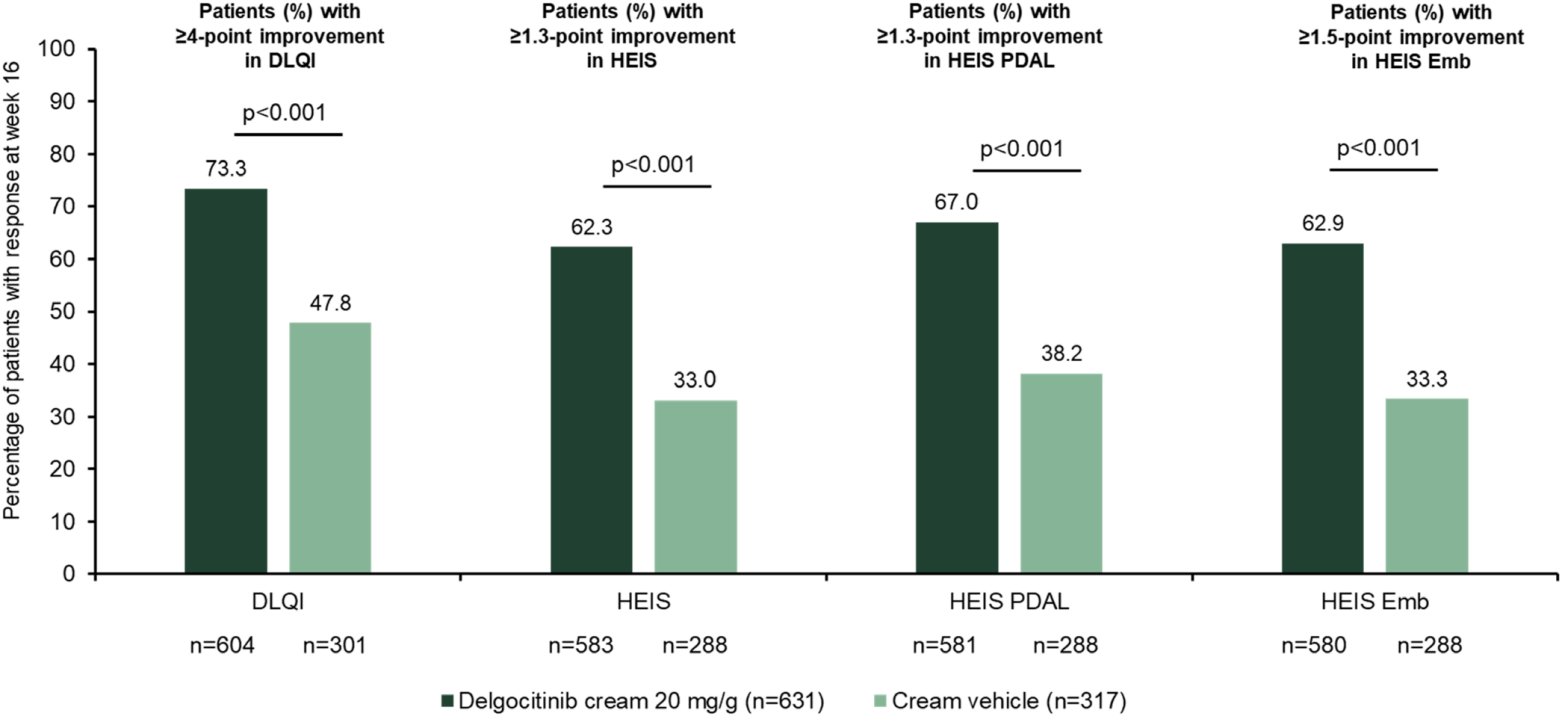
Proportions of patients achieving a minimum clinically important improvement in DLQI, HEIS, HEIS PDAL, and HEIS Emb after 16 weeks of treatment with delgocitinib cream or cream vehicle. DLQI, Dermatology Life Quality Index; Emb, Embarrassment; HEIS; Hand Eczema Impact Scale; PDAL, Proximal Daily Activity Limitations.

At week 16, more patients treated with delgocitinib cream than cream vehicle achieved a clinically meaningful response in DLQI (≥ 4 reduction) (73.3% [95% CI: 69.7, 76.7] vs. 47.8% [95% CI: 42.3, 53.5]; *p* < 0.001) (Figure 3). The estimated cumulative incidence of a DLQI response was also higher with delgocitinib cream (86.3% [95% CI: 83.3, 88.8] vs. 69.2% [95% CI: 63.6, 74.0]) (Figure S1). Treatment with delgocitinib cream was associated with a significantly longer adjusted mean time with a DLQI response (80.5 ± 1.5 versus 53.2 ± 2.3 days; *p* < 0.001) (Table S3). Median time to response was 21 days (95% CI: 10, 27) with delgocitinib cream and 30 days (95% CI: 28, 55) with cream vehicle. Among DLQI responders to delgocitinib cream, adjusted mean (SE) time in response from baseline to week 16 was 83.9 ± 1.1 days.

### 4.3 HEIS, HEIS PDAL and HEIS Emb

Treatment with delgocitinib cream resulted in significantly greater improvement in HEIS compared to cream vehicle at week 16 (LS mean change [SE] −1.46 ± 0.04 vs. −0.73 ± 0.05; treatment difference −0.72 [95% CI: −0.86, −0.59]; *p* < 0.001) (Figure 1C). Improvement in HEIS with delgocitinib cream was evident from week 1 onwards and continued to increase across the 16‐week period. Improvement in HEIS at week 16 was significantly greater with delgocitinib cream across all individual HEIS domains/items (Figure 2 and Table S2).

A significantly higher proportion of patients achieved a clinically important HEIS response (≥ 1.3‐point improvement) at week 16 with delgocitinib cream compared with cream vehicle (62.3% vs. 33.0%; *p* < 0.001) (Figure 3). Estimated cumulative incidence of HEIS response was also higher with delgocitinib cream at week 16 (76.5% [95% CI: 72.8, 79.7] vs. 54.9% [95% CI: 48.9, 60.4]) (Figure S1). The median time to response was 21 days (95% CI: 16, 27) with delgocitinib cream compared to 84 days (95% CI: 56, 119) with cream vehicle. Patients in the delgocitinib cream group spent a significantly longer adjusted mean time with a HEIS response (61.9 ± 1.7 vs. 33.4 ± 2.3 days; *p* < 0.001) (Table S2). Among patients who achieved a HEIS response with delgocitinib cream, adjusted mean (SE) time in HEIS response was 72.4 ± 1.4 days.

Results for HEIS PDAL and HEIS Emb were similar to those for HEIS, with significantly greater mean improvement from baseline (Figure 1) and more patients achieving a clinically important response (Figure 3, Table S3) with delgocitinib cream. Adjusted mean (SE) time in response from baseline to week 16 for delgocitinib cream compared with cream vehicle was 72.6 ± 1.3 days versus 58.0 ± 2.1 (*p* < 0.001) days for HEIS PDAL and 78.6 ± 1.4 days versus 61.0 ± 2.5 for HEIS Emb (both *p* < 0.001). Estimated cumulative incidence of HEIS PDAL and HEIS Emb response was also higher with delgocitinib cream at week 16 (Figure S1), with median times to HEIS PDAL and HEIS Emb response of 15 (95% CI: 14, 17) days and 31 (95% CI: 28, 56) days with delgocitinib cream and 29 (95% CI: 28, 56) and 163 (96% CI: 88, 168) days with cream vehicle, respectively.

**FIGURE 4:**
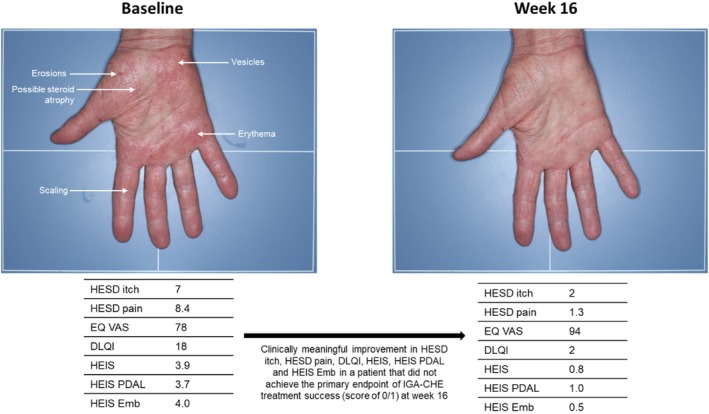
Improvement in EQ VAS, DLQI, HEIS, HEIS PDAL and HEIS Emb from baseline to week 16 in a patient with CHE (irritant, allergic, and atopic subtypes with a history of treatment with topical corticosteroids and topical calcineurin inhibitors) treated with delgocitinib cream. DLQI, Dermatology Life Quality Index; Emb, Embarrassment; HECSI, hand eczema severity index; HEIS; Hand Eczema Impact Scale HESD, hand eczema symptom diary; IGA‐CHE, Investigator's Global Assessment‐Chronic Hand Eczema; PDAL, Proximal Daily Activity Limitations; VAS, visual analogue scale.

A visual example of the improvements seen in EQ VAS, DLQI, and HEIS from baseline to week 16 in an individual patient treated with delgocitinib cream is shown in Figure 4. Of note, improvement in all PROs was seen despite no change in Investigator's Global Assessment for CHE (IGA‐CHE).

## 5 Discussion

In this pooled analysis of the DELTA 1 and 2 trials of patients with moderate to severe CHE, PRO scores at baseline demonstrated significant impairment of quality of life. Mean baseline EQ‐5D was 0.65, comparable to the impairment seen in patients with more generalised atopic dermatitis or psoriasis [27, 28]. Mean DLQI at baseline was 12.4, also indicating a very large effect on quality‐of‐life [24].

Twice‐daily application of delgocitinib cream 20 mg/g resulted in clinically and statistically significant improvement in HRQoL versus cream vehicle as assessed by all PRO measures. Strikingly, treatment differences in favour of delgocitinib cream were evident as early as week 1 and progressively increased over the 16‐week period. For EQ‐5D, delgocitinib cream improved all five domains, although as expected the benefit of treatment in terms of mobility was less evident. Delgocitinib cream showed a significant treatment effect on all 10 DLQI items, in particular skin‐related embarrassment or self‐consciousness, and effects on work, social and leisure, and other daily activities. The smallest treatment effect on DLQI items was seen in response to whether CHE had prevented work or study whereas the largest treatment effect was seen on ability to work or study. With regard to HEIS, all items showed significant improvement with delgocitinib cream.

All HRQoL measures reported that significantly more patients achieved clinically important improvement with delgocitinib cream and spent more time in an improved HRQoL state across the 16‐week period compared with cream vehicle. Mean times spent in response were higher with delgocitinib cream, indicating that responses were more sustained than with cream vehicle, an important consideration in a chronic disease.

Significantly greater improvements in PROs in patients treated with delgocitinib cream were observed despite improvements also being reported in the cream vehicle group. For example, mean change from baseline in DLQI approached the MCID in the cream vehicle group and nearly half the cream vehicle group (48%) had a DLQI response at week 16. The cream vehicle used in DELTA 1 and 2 was specially formulated for CHE and its regular application resulted in clinical benefit [22]. This, together with the effects of trial participation, may have had a beneficial impact on PROs.

PROs are an important complement to physician‐assessed endpoints in dermatologic diseases [29]. However, there is no consensus on which HRQoL instrument is most appropriate in CHE [30]. Although EQ‐5D does provide a more holistic evaluation of disease status and allows for comparison between different diseases, it is generic and not dermatology‐specific. However, its validity and responsiveness were reported to be good in people with skin diseases, with moderate to strong correlation between EQ‐5D and other skin‐specific HRQoL measures [28]. This observation was largely based on studies of patients with plaque psoriasis or psoriatic arthritis. DLQI is skin‐specific, and the most frequently used HRQoL measure in dermatology clinical trials and in the clinic [31]. The use of DLQI has been recommended by the EADV Task Forces on Quality of Life and Patient Oriented Outcomes and Occupational Skin Disease [32], and by the Harmonising Outcome Measures for Eczema (HOME) initiative for atopic eczema trials [33]. However, DLQI may not adequately address all aspects of HRQoL that are relevant to people with CHE, with aspects of physical functioning (e.g., touching or holding objects), daily living activities (e.g., housework that involves getting hands wet), and self‐care all being absent [26]. The localised nature of CHE may also limit the ability of DLQI to detect change [29]. It is interesting that this study identified significant improvement in both EQ‐5D and DLQI, re‐emphasising its high impact nature. The third instrument used, HEIS, was specifically designed to address some of the limitations of more general tools and to assess multidimensional aspects of HRQoL associated with CHE. HEIS was initially developed based on the literature and concept elicitation interviews with dermatologists and patients with CHE. Psychometric validity was then confirmed using data from a phase 2b trial of delgocitinib cream versus vehicle [26]. The data reported here represent the first use of HEIS in phase 3 studies with a large, multinational population and suggest strong utility in this cohort. All three HRQoL tools used in the DELTA 1 and 2 trials were generally consistent, suggesting all may have a role in determining the impact on HRQoL and the effects of treatment in patients with moderate to severe CHE.

Limitations of this analysis include a predominately white trial population with a short duration of 16 weeks treatment. Assessment of the effects of delgocitinib cream on HRQoL in more racially and ethnically diverse populations is required. The longer‐term (up to 52 weeks) impact of delgocitinib cream on HRQoL is being investigated in the DELTA 3 trial [34].

In conclusion, twice‐daily treatment with delgocitinib cream 20 mg/g in patients with moderate to severe CHE resulted in a clinically and statistically significant improvement in HRQoL versus cream vehicle, as measured by generic, dermatology‐specific, and CHE‐specific measures. Given that HEIS was specifically developed to assess HRQoL in patients with CHE, it may have greater utility in clinical trials in CHE than more generic tools. However, both EQ‐5D and DLQI were also shown to correlate with clinical improvement, and all three measures should be considered complementary.

## Author Contributions

**A.B.:** conceptualization, investigation, validation, writing – review and editing. **L.G.:** investigation, writing‐review and editing. **R.W.:** investigation, writing‐review and editing. **M.L.S.:** investigation, validation, writing – review and editing. **E.D.A.:** conceptualization, writing – review and editing. **D.M.:** conceptualization, writing – review and editing. **N.N.:** conceptualization, writing – review and editing. **H.T.:** formal analysis, writing – review and editing.

## Funding

This work was supported by LEO Pharma A/S, Ballerup, Denmark.

## Ethics Statement

Both trials were conducted in accordance with the Declaration of Helsinki, Good Clinical Practice guidelines, and applicable regulatory requirements, and were approved by the local institutional review board or independent ethics committee of each institution. All patients provided written informed consent before undergoing any study‐related procedures.

## Conflicts of Interest

**A.B**. has been a speaker/advisor/investigator and/or received research funding from AbbVie, Almirall, Amgen, AstraZeneca, Biocryst, Biofrontera, CSL Behring, Eli Lilly, Escient, Galderma, Genentech, Gilead, Incyte, Jannsen, Jasper, Kalvista, LEO Pharma A/S, L'Oréal, Novartis, Otsuka, Pierre Fabre, Pfizer, Pharvaris, Regeneron, Sanofi and Takeda. **L.G**. has been a consultant for Abbvie, Amgen, Aslan, Astellas, Bausch Health, BMS, Celgene, Cipher, Elli Lilly, Galderma, GSK, Janssen, Johnson & Johnson, La Roche Posay, Leo Pharma, Merck, Novartis, Pfizer, Pierre Fabre, and Sanofi Aventis, an investigator for Abbvie, Astellas, Amgen, Bausch Health, BMS, Celgene, Cipher, Elli Lilly, Galderma, GSK, Janssen, La Roche Posay, Leo Pharma, Novartis, Pfizer, Roche and Sun Pharmaceuticals, a speaker for Abbvie, Amgen, Astellas, Bausch Health, Celgene, Cipher, Elli Lilly, Galderma, GSK, Janssen, Johnson & Johnson, La Roche Posay, Leo Pharma, Novartis, Pfizer, Pierre Fabre, Sanofi Aventis and Sun Pharmaceuticals. **R.W**. has been a consultant, advisory board member, investigator, and/or speaker for AbbVie, Almirall, Amgen, Boehringer Ingelheim, Bristol Myers Squibb, Celgene, Eli Lilly, Galderma Incyte, Janssen‐Cilag, LEO Pharma A/S, Novartis, Pfizer, Sanofi, and UCB. **M.L.S**. has been a consultant, advisory board member, investigator, and/or speaker for AbbVie, Amgen, Galderma Incyte, LEO Pharma A/S, Pfizer, Regeneron Pharmaceuticals Inc. and Sanofi Genzyme. **E.D.A., D.M., N.N**., and **H.T**. are shareholders and employees of LEO Pharma A/S.

## Supporting information


**Table S1:** Description of scores used to assess health‐related quality of life
**Table S2:** EQ‐5D, DLQI and HEIS at baseline and 16 weeks.
**Table S3:** Mean area under the curve from baseline to week 16 for clinically significant improvement in DLQI, HEIS, HEIS PDAL and HEIS Emb with delgocitinib cream or cream vehicle for all patients and for patients achieving improvement
**Figure S1:** Estimated cumulative incidence of patients achieving a minimum clinically important improvement in (A) DLQI, (B) HEIS (C) HEIS PDAL and (D) HEIS Emb over 16 weeks of treatment with delgocitinib cream or cream vehicle.
**Data S1:** CONSORT_2025_editable_checklist.

## Data Availability

The data that support the findings of this trial are available on request. All requests are reviewed independently of LEO Pharma by an external Patient and Scientific Review Board. The data are not publicly available due to privacy or ethical restrictions.
