# Supplementary material for: Effect of Delgocitinib Cream on Health‐Related Quality of Life in Patients With Moderate to Severe Chronic Hand Eczema
Source: Contact Dermatitis. 2026 Mar 3;95(1):91–9. doi: 10.1111/cod.70114 (PMC13238450; doi:10.1111/cod.70114)
Supplement: Supplementary file 1 — Table S1: Description of scores used to assess health‐related quality of life Table S2: EQ‐5D, DLQI and HEIS at baseline and 16 weeks. Table S3: Mean area under the curve from baseline to week 16 for clinically significant improvement in DLQI, HEIS, HEIS PDAL and HEIS Emb with delgocitinib cream or cream vehicle for all patients and for patients achieving improvement Figure S1: Estimated cumulative incidence of patients achieving a minimum clinically important improvement in (A) DLQI, (B) HEIS (C) HEIS PDAL and (D) HEIS Emb over 16 weeks of treatment with delgocitinib cream or cream vehicle. Data S1: CONSORT_2025_editable_checklist. [file COD-95-91-s001.docx]

**Effect of delgocitinib cream on health-related quality of life in patients with moderate to severe Chronic Hand Eczema**

***Andrea Bauer,^1^ Lyn Guenther,^2^ Richard Woolf,^3^ Eydna Didriksen Apol^4^, Douglas Maslin^4^, Nanna Nyholm,^4^ Henrik Thoning,^4^ Marie Louise Schuttelaar^5^***

*^1^Department of Dermatology, University Allergy Centre, University Hospital Carl Gustav Carus, Technical University, Dresden, Germany; ^2^Division of Dermatology, Department of Medicine, Western University, London, ON, Canada; ^3^St John's Institute of Dermatology, Guy's and St Thomas' NHS Foundation Trust , London, UK; ^4^LEO Pharma A/S, Ballerup, Denmark; ^5^Department of Dermatology, University Medical Center Groningen, University of Groningen, Groningen, The Netherlands.*

**Supplementary material**

**Table S1.** Description of scores used to assess health-related quality of life

**Table S2.** EQ-5D, DLQI and HEIS at baseline and 16 weeks.

**Table S3.** Mean area under the curve from baseline to week 16 for clinically significant improvement in DLQI, HEIS, HEIS PDAL and HEIS Emb with delgocitinib cream or cream vehicle for all patients and for patients achieving improvement

**Figure S1.** Estimated cumulative incidence of patients achieving a minimum clinically important improvement in (A) DLQI, (B) HEIS (C) HEIS PDAL and (D) HEIS Emb over 16 weeks of treatment with delgocitinib cream or cream vehicle.**Table S1.** Description of patient-reported outcomes

**Table S1.** Description of scores used to assess health-related quality of life

|  | **Description** | **Disease area** | **Recall period** | **Range/**  **interpretation** | **Assessed at:** | **MCID** |
| --- | --- | --- | --- | --- | --- | --- |
| EQ-5D^1^ | EQ-5D consists of two parts: a questionnaire with 5 dimensions: mobility, self-care, usual activities, pain/discomfort and anxiety/depression. Each dimension has 5 levels: 1, no problems; 2, slight problems; 3, moderate problems; 4, severe problems; and 5, extreme problems. The digits for the 5 dimensions are combined in a  5-digit code (with no arithmetic properties). These profiles have then been applied different preferences, where members of the general public have been asked to imagine living in health states described by the EQ-5D descriptive system. This then generates a score from 0 (death) to 1 (perfect health).  The second part is the EQ VAS, which captures the respondent’s overall assessment of their health on a scale from 0 (worst health imaginable) to 100 (best health imaginable) | Generic | Same day | 5-digit code, from which a single index value can be derived  EQ VAS from 0 (worst health imaginable) to 100 (best health imaginable), so a low score is indicative of poor quality of life | Baseline and weeks 1, 4, 8, 12 and 16 | Not applicable |
| Dermatology Life Quality Index (DLQI)^2^ | Ten questions to assesses the impact of skin disease on symptoms, feelings, daily activities, leisure, work and school, personal relationships and the impact of treatment. Each question has 4 levels: 0, not at all; 1 a little; 2, a lot; and 3, very much | Dermatology-specific | 7 days | 0–30, with a high score indicative of poor quality of life | Baseline and weeks 1, 4, 8, 12 and 16 | ≥4-point improvement (Hongbo 2005) |
| Hand Eczema Impact Scale (HEIS)^3^ | Nine questions to assess the impact of CHE grouped into 5 domains: proximal daily activity limitations (PDAL; 3 items), embarrassment with the appearance of hands (Emb; 2 items), frustration with CHE (1 item), sleep (1 item), work (1 item) and grip (1 item). Each item has 5 levels: 0, not at all; 1, a little; 2, moderately; 3, a lot; and 4, extremely | CHE-specific | 7 days | 0–4, with total score an average of all 9 items and a high score representing a high disease impact | Baseline and weeks 1, 2, 4, 8, 12 and 16 | ≥1.3-point improvement |
| HEIS Proximal Daily Activity Limitations (PDAL)^3^ | Subset of HEIS (3 items - ability to use soaps/cleaning products, difficulty in doing housework that involves getting hands wet, difficulty in washing) | CHE-specific | 7 days | 0–4, with total score an average of all 3 items | Baseline and weeks 1, 2, 4, 8, 12 and 16 | ≥1.3-point improvement |
| HEIS Embarrassment (Emb)^3^ | Subset of HEIS (2 items - how embarrassed about how hands look, how much appearance of hands is disliked) | CHE-specific | 7 days | 0–4, with total score an average of both items | Baseline and weeks 1, 2, 4, 8, 12 and 16 | ≥1.5-point improvement |
| CHE, Chronic Hand Eczema; DLQI, Dermatology Life Quality Index; Emb, Embarrassment; HEIS; Hand Eczema Impact Scale; HESD, Hand Eczema Symptom Diary MCID, minimum clinically important difference; PDAL, Proximal Daily Activity Limitations | | | | | | |
| 1. EuroQol Research Foundation. EQ-5D-5L User Guide. Version 3. September 2019. https://euroqol.org/wp-content/uploads/2023/11/EQ-5D-5LUserguide-23-07.pdf. Accessed March 2025; 2. Finlay AY, Khan GK. Dermatology Life Quality Index (DLQI)--a simple practical measure for routine clinical use. Clin Exp Dermatol. 1994;19:210-6;3. Weisshaar E, Yüksel YT, Agner T, Larsen LS, Grant L, Arbuckle R, et al. Development and Validation of a Patient-Reported Outcome Measure of the Impact of Chronic Hand Eczema on Health-Related Quality of Life: the Hand Eczema Impact Scale (HEIS). Dermatol Ther (Heidelb). 2024;14(11):3047-3070. | | | | | | |

**Table S2.** EQ-5D, DLQI and HEIS at baseline and 16 weeks

| **EQ-5D** | | | | | | |
| --- | --- | --- | --- | --- | --- | --- |
|  | **Delgocitinib cream 20 mg/g (N=631)** | | **Cream vehicle (N=317)** | |  |  |
|  | **Baseline** | **Week 16** | **Baseline** | **Week 16** | **Difference in percentage (95% CI)** | **p-value** |
| **Mobility** | | | | | | |
| I have no problems in walking about | 544.0 (86.2) | 564.0 (89.4) | 274.0 (86.4) | 265.0 (83.6) | 6.3 (2.1, 10.5) | 0.002 |
| I have slight problems in walking about | 46.0 (7.3) | 42.0 (6.7) | 26.0 (8.2) | 30.0 (9.5) | −3.0 (−6.7, 0.6) | 0.081 |
| I have moderate problems in walking about | 25.0 (4.0) | 13.0 (2.1) | 11.0 (3.5) | 15.0 (4.7) | −2.8 (−5.2, −0.3) | 0.009 |
| I have severe problems in walking about | 13.0 (2.1) | 12.0 (1.9) | 5.0 (1.6) | 6.0 (1.9) | −0.3 (−1.9, 1.4) | 0.754 |
| I am unable to walk about | 3.0 (0.5) | 0.0 (0.0) | 1.0 (0.3) | 1.0 (0.3) | −0.2 (−0.8, 0.3) | 0.317 |
| **Self** | | | | | | |
| I have no problems washing or dressing myself | 438.0 (69.4) | 529.0 (83.8) | 214.0 (67.5) | 215.0 (67.8) | 15.3 (10.0, 20.6) | <0.001 |
| I have slight problems washing or dressing myself | 116.0 (18.4) | 76.0 (12.0) | 55.0 (17.4) | 67.0 (21.1) | −8.2 (−13.2, −3.3) | <0.001 |
| I have moderate problems washing or dressing myself | 60.0 (9.5) | 17.0 (2.7) | 40.0 (12.6) | 27.0 (8.5) | −5.5 (−8.7, −2.2) | <0.001 |
| I have severe problems washing or dressing myself | 16.0 (2.5) | 8.0 (1.3) | 7.0 (2.2) | 7.0 (2.2) | −1.3 (−3.1, 0.4) | 0.093 |
| I am unable to wash or dress myself | 1.0 (0.2) | 1.0 (0.2) | 1.0 (0.3) | 1.0 (0.3) | −0.3 (−0.9, 0.3) | 0.234 |
| **Usual Activities** | | | | | | |
| I have no problems doing my usual activities | 244.0 (38.7) | 423.0 (67.0) | 125.0 (39.4) | 154.0 (48.6) | 18.8 (12.5, 25.0) | <0.001 |
| I have slight problems doing my usual activities | 174.0 (27.6) | 140.0 (22.2) | 90.0 (28.4) | 98.0 (30.9) | −9.2 (−15.2, −3.3) | 0.002 |
| I have moderate problems doing my usual activities | 139.0 (22.0) | 41.0 (6.5) | 76.0 (24.0) | 49.0 (15.5) | −8.1 (−12.4, −3.8) | <0.001 |
| I have severe problems doing my usual activities | 70.0 (11.1) | 25.0 (4.0) | 23.0 (7.3) | 14.0 (4.4) | −1.1 (−3.7, 1.5) | 0.395 |
| I am unable to do my usual activities | 4.0 (0.6) | 2.0 (0.3) | 3.0 (0.9) | 2.0 (0.6) | −0.3 (−1.3, 0.7) | 0.419 |
| **Pain/Discomfort** | | | | | | |
| I have no pain or discomfort | 64.0 (10.1) | 270.0 (42.8) | 38.0 (12.0) | 72.0 (22.7) | 20.5 (14.6, 26.4) | <0.001 |
| I have slight pain or discomfort | 166.0 (26.3) | 231.0 (36.6) | 80.0 (25.2) | 107.0 (33.8) | 2.5 (−4.0, 9.0) | 0.449 |
| I have moderate pain or discomfort | 253.0 (40.1) | 87.0 (13.8) | 110.0 (34.7) | 90.0 (28.4) | −15.5 (−21.2, −9.8) | <0.001 |
| I have severe pain or discomfort | 123.0 (19.5) | 35.0 (5.5) | 84.0 (26.5) | 43.0 (13.6) | −6.9 (−11.0, −2.9) | <0.001 |
| I have extreme pain or discomfort | 25.0 (4.0) | 8.0 (1.3) | 5.0 (1.6) | 5.0 (1.6) | −0.5 (−2.1, 1.0) | 0.472 |
| **Anxiety/Depression** | | | | | | |
| I am not anxious or depressed | 318.0 (50.4) | 424.0 (67.2) | 147.0 (46.4) | 144.0 (45.4) | 19.3 (13.4, 25.3) | <0.001 |
| I am slightly anxious or depressed | 170.0 (26.9) | 148.0 (23.5) | 80.0 (25.2) | 95.0 (30.0) | −6.3 (−12.2, −0.3) | 0.035 |
| I am moderately anxious or depressed | 97.0 (15.4) | 41.0 (6.5) | 57.0 (18.0) | 52.0 (16.4) | −9.1 (−13.5, −4.7) | <0.001 |
| I am severely anxious or depressed | 41.0 (6.5) | 14.0 (2.2) | 28.0 (8.8) | 20.0 (6.3) | −3.4 (−6.2, −0.6) | 0.008 |
| I am extremely anxious or depressed | 5.0 (0.8) | 4.0 (0.6) | 5.0 (1.6) | 6.0 (1.9) | −0.5 (−1.9, 0.8) | 0.397 |

| **DLQI** | | | | | | |
| --- | --- | --- | --- | --- | --- | --- |
|  | **Delgocitinib cream 20 mg/g** | | **Cream vehicle** | |  | |
|  | **Baseline, (N=631)** | **Week 16, (N=631)** | **Baseline, (N=317)** | **Week 16, (N=317)** | **Difference in percentage (95% CI)** | **p-value** |
| How Itchy, Sore, Painful, Stinging | | | | | | |
| Not at all | 1.0 (0.2) | 125.0 (19.8) | 1.0 (0.3) | 28.0 (8.8) | 11.0 (6.6, 15.5) | <0.001 |
| A little | 62.0 (9.8) | 298.0 (47.2) | 46.0 (14.5) | 101.0 (31.9) | 15.5 (9.0, 21.9) | <0.001 |
| A lot | 284.0 (45.0) | 137.0 (21.7) | 125.0 (39.4) | 83.0 (26.2) | −4.5 (−10.4, 1.3) | 0.120 |
| Very much | 284.0 (45.0) | 71.0 (11.3) | 145.0 (45.7) | 105.0 (33.1) | −22.0 (−27.7, −16.3) | <0.001 |
| How Embarrassed, Self-Conscious | | | | | | |
| Not at all | 40.0 (6.3) | 295.0 (46.8) | 21.0 (6.6) | 76.0 (24.0) | 22.7 (16.8, 28.6) | <0.001 |
| A little | 176.0 (27.9) | 204.0 (32.3) | 100.0 (31.5) | 105.0 (33.1) | −0.8 (−7.2, 5.6) | 0.803 |
| A lot | 247.0 (39.1) | 91.0 (14.4) | 103.0 (32.5) | 72.0 (22.7) | −8.6 (−14.0, −3.3) | <0.001 |
| Very much | 168.0 (26.6) | 41.0 (6.5) | 93.0 (29.3) | 64.0 (20.2) | −13.3 (−18.0, −8.5) | <0.001 |
| Interfered Shopping, Home, Yard | | | | | | |
| Not at all | 56.0 (8.9) | 300.0 (47.5) | 23.0 (7.3) | 82.0 (25.9) | 21.4 (15.4, 27.5) | <0.001 |
| A little | 199.0 (31.5) | 187.0 (29.6) | 99.0 (31.2) | 108.0 (34.1) | −4.4 (−10.9, 2.0) | 0.164 |
| A lot | 231.0 (36.6) | 69.0 (10.9) | 113.0 (35.6) | 65.0 (20.5) | −9.5 (−14.6, −4.5) | <0.001 |
| Very much | 140.0 (22.2) | 30.0 (4.8) | 73.0 (23.0) | 40.0 (12.6) | −7.5 (−11.4, −3.7) | <0.001 |
| Influenced Clothes You Wear | | | | | | |
| Not at all | 263.0 (41.7) | 428.0 (67.8) | 109.0 (34.4) | 155.0 (48.9) | 17.3 (10.9, 23.8) | <0.001 |
| A little | 190.0 (30.1) | 100.0 (15.8) | 99.0 (31.2) | 73.0 (23.0) | −5.9 (−11.3, −0.5) | 0.027 |
| A lot | 94.0 (14.9) | 28.0 (4.4) | 54.0 (17.0) | 38.0 (12.0) | −7.3 (−11.2, −3.5) | <0.001 |
| Very much | 31.0 (4.9) | 10.0 (1.6) | 23.0 (7.3) | 15.0 (4.7) | −2.7 (−5.1, −0.3) | 0.013 |
| Affected Social, Leisure Activity | | | | | | |
| Not at all | 154.0 (24.4) | 408.0 (64.7) | 87.0 (27.4) | 135.0 (42.6) | 21.7 (15.3, 28.1) | <0.001 |
| A little | 241.0 (38.2) | 131.0 (20.8) | 110.0 (34.7) | 89.0 (28.1) | −7.3 (−13.2, −1.4) | 0.012 |
| A lot | 149.0 (23.6) | 34.0 (5.4) | 70.0 (22.1) | 55.0 (17.4) | −11.9 (−16.4, −7.5) | <0.001 |
| Very much | 59.0 (9.4) | 17.0 (2.7) | 40.0 (12.6) | 19.0 (6.0) | −3.0 (−5.8, −0.1) | 0.020 |
| Made It Difficult to Do Any Sports | | | | | | |
| Not at all | 157.0 (24.9) | 364.0 (57.7) | 73.0 (23.0) | 111.0 (35.0) | 22.2 (15.8, 28.7) | <0.001 |
| A little | 175.0 (27.7) | 99.0 (15.7) | 82.0 (25.9) | 71.0 (22.4) | −6.4 (−11.7, −1.2) | 0.013 |
| A lot | 122.0 (19.3) | 35.0 (5.5) | 75.0 (23.7) | 41.0 (12.9) | −6.9 (−10.9, −3.0) | <0.001 |
| Very much | 57.0 (9.0) | 17.0 (2.7) | 27.0 (8.5) | 23.0 (7.3) | −4.5 (−7.4, −1.6) | <0.001 |
| Prevented Working or Studying | | | | | | |
| No | 400.0 (63.4) | 479.0 (75.9) | 207.0 (65.3) | 219.0 (69.1) | 6.8 (1.0, 12.8) | 0.020 |
| Yes | 130.0 (20.6) | 52.0 (8.2) | 62.0 (19.6) | 46.0 (14.5) | −6.3 (−10.3, −2.3) | <0.001 |
| Problem at Work or Studying | | | | | | |
| Not at all | 113.0 (28.3) | 259.0 (71.5) | 67.0 (32.4) | 89.0 (48.1) | 24.7 (16.5, 32.8) | <0.001 |
| A little | 198.0 (49.5) | 87.0 (24.0) | 91.0 (44.0) | 74.0 (40.0) | −17.1 (−25.4, −8.8) | <0.001 |
| A lot | 89.0 (22.3) | 16.0 (4.4) | 49.0 (23.7) | 22.0 (11.9) | −7.6 (−12.6, −2.5) | <0.001 |
| Problem Partner, Friends, Relative | | | | | | |
| Not at all | 243.0 (38.5) | 447.0 (70.8) | 121.0 (38.2) | 165.0 (52.1) | 18.2 (11.9, 24.4) | <0.001 |
| A little | 216.0 (34.2) | 101.0 (16.0) | 100.0 (31.5) | 78.0 (24.6) | −8.6 (−14.2, −3.1) | 0.001 |
| A lot | 102.0 (16.2) | 18.0 (2.9) | 53.0 (16.7) | 31.0 (9.8) | −6.9 (−10.4, −3.5) | <.0001 |
| Very much | 39.0 (6.2) | 13.0 (2.1) | 26.0 (8.2) | 18.0 (5.7) | −3.0 (−5.5, −0.4) | 0.010 |
| Caused Any Sexual Difficulties | | | | | | |
| Not at all | 264.0 (41.8) | 413.0 (65.5) | 148.0 (46.7) | 154.0 (48.6) | 17.0 (10.3, 23.6) | <0.001 |
| A little | 149.0 (23.6) | 68.0 (10.8) | 59.0 (18.6) | 61.0 (19.2) | −9.7 (−14.5, −5.0) | <0.001 |
| A lot | 81.0 (12.8) | 21.0 (3.3) | 40.0 (12.6) | 26.0 (8.2) | −4.8 (−8.0, −1.6) | <0.001 |
| Very much | 23.0 (3.6) | 10.0 (1.6) | 18.0 (5.7) | 11.0 (3.5) | −1.3 (−3.4, 0.8) | 0.167 |
| How Much a Problem is Treatment | | | | | | |
| Not at all | 159.0 (25.2) | 376.0 (59.6) | 79.0 (24.9) | 146.0 (46.1) | 14.1 (7.6, 20.5) | <0.001 |
| A little | 213.0 (33.8) | 182.0 (28.8) | 112.0 (35.3) | 97.0 (30.6) | −2.3 (−8.5, 3.8) | 0.453 |
| A lot | 136.0 (21.6) | 31.0 (4.9) | 46.0 (14.5) | 43.0 (13.6) | −9.4 (−13.4, −5.3) | <0.001 |
| Very much | 51.0 (8.1) | 18.0 (2.9) | 35.0 (11.0) | 17.0 (5.4) | −2.0 (−4.7, 0.7) | 0.125 |
| CI = confidence interval., WOCF = worst observation carried forward. N = number of subjects with data available., % = percentage of subjects. Composite estimand: Data considered nonresponse by using WOCF (including the baseline value) after initiation ofrescue treatment or after permanent discontinuation of IMP. Missing data imputed using WOCF (including the baseline value). Cochran-Mantel-Haenszel test, stratified by study ID, region, baseline IGA-CHE and baseline DLQI dimension response. | | | | | | |

| **HEIS** | | | | | | |
| --- | --- | --- | --- | --- | --- | --- |
|  | **Delgocitinib cream 20 mg/g** | | **Cream vehicle** | |  | |
|  | **Baseline, (N=631)** | **Week 16, (N=638)** | **Baseline, (N=317)** | **Week 16, (N=321)** | **Difference in percentage (95% CI)** | **p−value** |
| Over the past seven days, how much has your hand eczema impacted on your ability to use soaps/cleaning products? | | | | | | |
| Not at all | 18.0 (2.9) | 210.0 (32.9) | 8.0 (2.5) | 49.0 (15.3) | 18.0 (12.8, 23.3) | <0.001 |
| A little | 63.0 (10.0) | 209.0 (32.8) | 42.0 (13.2) | 78.0 (24.3) | 7.4 (1.3, 13.4) | 0.020 |
| Moderate | 166.0 (26.3) | 101.0 (15.8) | 70.0 (22.1) | 69.0 (21.5) | −5.7 (−11.1, −0.4) | 0.031 |
| A lot | 236.0 (37.4) | 89.0 (13.9) | 125.0 (39.4) | 75.0 (23.4) | −9.2 (−14.6, −3.9) | <0.001 |
| Extremely | 148.0 (23.5) | 29.0 (4.5) | 72.0 (22.7) | 50.0 (15.6) | −10.4 (−14.6, −6.3) | <0.001 |
| Over the past seven days, how much has your hand eczema made it hard to do housework that involved your hands getting wet (e.g. washing dishes)? | | | | | | |
| Not at all | 15.0 (2.4) | 204.0 (32.0) | 7.0 (2.2) | 54.0 (16.8) | 16.2 (10.8, 21.5) | <0.001 |
| A little | 35.0 (5.5) | 200.0 (31.3) | 28.0 (8.8) | 64.0 (19.9) | 10.5 (4.7, 16.3) | <0.001 |
| Moderate | 113.0 (17.9) | 111.0 (17.4) | 61.0 (19.2) | 62.0 (19.3) | −2.6 (−8.0, 2.7) | 0.326 |
| A lot | 252.0 (39.9) | 78.0 (12.2) | 101.0 (31.9) | 72.0 (22.4) | −11.1 (−16.5, −5.8) | <0.001 |
| Extremely | 216.0 (34.2) | 45.0 (7.1) | 120.0 (37.9) | 69.0 (21.5) | −12.9 (−17.7, −8.2) | <0.001 |
| Over the past seven days, how much has your hand eczema made it hard to wash yourself? | | | | | | |
| Not at all | 85.0 (13.5) | 373.0 (58.5) | 36.0 (11.4) | 116.0 (36.1) | 21.3 (15.0, 27.7) | <0.001 |
| A little | 105.0 (16.6) | 136.0 (21.3) | 61.0 (19.2) | 72.0 (22.4) | −1.5 (−7.1, 4.2) | 0.609 |
| Moderate | 222.0 (35.2) | 79.0 (12.4) | 96.0 (30.3) | 59.0 (18.4) | −6.2 (−11.3, −1.2) | 0.010 |
| A lot | 156.0 (24.7) | 30.0 (4.7) | 87.0 (27.4) | 52.0 (16.2) | −11.0 (−15.4, −6.7) | <0.001 |
| Extremely | 63.0 (10.0) | 20.0 (3.1) | 37.0 (11.7) | 22.0 (6.9) | −2.6 (−5.4, 0.2) | 0.050 |
| Over the past seven days, how embarrassed have you felt about how your hands look due to your hand eczema? | | | | | | |
| Not at all | 45.0 (7.1) | 303.0 (47.5) | 19.0 (6.0) | 66.0 (20.6) | 26.2 (20.3, 32.1) | <0.001 |
| A little | 85.0 (13.5) | 151.0 (23.7) | 35.0 (11.0) | 78.0 (24.3) | −1.7 (−7.5, 4.2) | 0.576 |
| Moderate | 136.0 (21.6) | 78.0 (12.2) | 75.0 (23.7) | 58.0 (18.1) | −5.4 (−10.4, −0.4) | 0.029 |
| A lot | 204.0 (32.3) | 67.0 (10.5) | 103.0 (32.5) | 67.0 (20.9) | −9.8 (−14.8, −4.7) | <0.001 |
| Extremely | 161.0 (25.5) | 39.0 (6.1) | 85.0 (26.8) | 52.0 (16.2) | −9.4 (−13.8, −5.1) | <0.001 |
| Over the past seven days, how much have you disliked the appearance of your hands? | | | | | | |
| Not at all | 16.0 (2.5) | 199.0 (31.2) | 13.0 (4.1) | 39.0 (12.1) | 18.3 (13.2, 23.3) | <0.001 |
| A little | 35.0 (5.5) | 193.0 (30.3) | 19.0 (6.0) | 61.0 (19.0) | 10.9 (5.3, 16.6) | <0.001 |
| Moderate | 105.0 (16.6) | 87.0 (13.6) | 54.0 (17.0) | 62.0 (19.3) | −5.3 (−10.5, −0.1) | 0.036 |
| A lot | 241.0 (38.2) | 106.0 (16.6) | 98.0 (30.9) | 75.0 (23.4) | −7.3 (−12.9, −1.7) | 0.008 |
| Extremely | 234.0 (37.1) | 53.0 (8.3) | 133.0 (42.0) | 84.0 (26.2) | −16.6 (−21.8, −11.4) | <0.001 |
| Over the past seven days, how much has your hand eczema made you feel frustrated? | | | | | | |
| Not at all | 13.0 (2.1) | 263.0 (41.2) | 12.0 (3.8) | 71.0 (22.1) | 18.4 (12.6, 24.2) | <0.001 |
| A little | 63.0 (10.0) | 172.0 (27.0) | 28.0 (8.8) | 64.0 (19.9) | 6.5 (0.8, 12.2) | 0.030 |
| Moderate | 156.0 (24.7) | 88.0 (13.8) | 68.0 (21.5) | 38.0 (11.8) | 1.7 (−2.8, 6.2) | 0.465 |
| A lot | 220.0 (34.9) | 74.0 (11.6) | 111.0 (35.0) | 69.0 (21.5) | −9.7 (−14.9, −4.5) | <0.001 |
| Extremely | 179.0 (28.4) | 41.0 (6.4) | 98.0 (30.9) | 79.0 (24.6) | −16.8 (−21.8, −11.9) | <0.001 |
| Over the past seven days, how much has your hand eczema impacted on the quality of your sleep? | | | | | | |
| Not at all | 72.0 (11.4) | 406.0 (63.6) | 47.0 (14.8) | 118.0 (36.8) | 27.1 (20.9, 33.4) | <0.001 |
| A little | 184.0 (29.2) | 124.0 (19.4) | 97.0 (30.6) | 82.0 (25.5) | −6.8 (−12.6, −1.0) | 0.016 |
| Moderate | 220.0 (34.9) | 73.0 (11.4) | 93.0 (29.3) | 63.0 (19.6) | −8.9 (−13.9, −3.8) | <0.001 |
| A lot | 130.0 (20.6) | 24.0 (3.8) | 62.0 (19.6) | 41.0 (12.8) | −8.7 (−12.6, −4.8) | <0.001 |
| Extremely | 25.0 (4.0) | 11.0 (1.7) | 18.0 (5.7) | 17.0 (5.3) | −2.7 (−5.3, −0.2) | 0.017 |
| Over the past seven days, how much has your hand eczema made it hard to work (e.g., paid work or voluntary)? | | | | | | |
| Not at all | 65.0 (10.3) | 379.0 (59.4) | 44.0 (13.9) | 113.0 (35.2) | 23.5 (17.3, 29.7) | <0.001 |
| A little | 105.0 (16.6) | 127.0 (19.9) | 53.0 (16.7) | 66.0 (20.6) | −1.7 (−7.3, 3.8) | 0.530 |
| Moderate | 202.0 (32.0) | 57.0 (8.9) | 78.0 (24.6) | 55.0 (17.1) | −9.2 (−14.0, −4.4) | <0.001 |
| A lot | 170.0 (26.9) | 51.0 (8.0) | 83.0 (26.2) | 63.0 (19.6) | −10.4 (−15.1, −5.6) | <0.001 |
| Extremely | 89.0 (14.1) | 24.0 (3.8) | 59.0 (18.6) | 24.0 (7.5) | −2.2 (−5.2, 0.8) | 0.121 |
| Over the past seven days, how much has your hand eczema made it hard to grip or hold objects (e.g. a mobile phone, pen or pencil)? | | | | | | |
| Not at all | 69.0 (10.9) | 361.0 (56.6) | 38.0 (12.0) | 103.0 (32.1) | 23.6 (17.4, 29.8) | <0.001 |
| A little | 112.0 (17.7) | 118.0 (18.5) | 51.0 (16.1) | 70.0 (21.8) | −4.2 (−9.8, 1.3) | 0.127 |
| Moderate | 183.0 (29.0) | 84.0 (13.2) | 80.0 (25.2) | 60.0 (18.7) | −5.7 (−10.9, −0.6) | 0.022 |
| A lot | 185.0 (29.3) | 57.0 (8.9) | 99.0 (31.2) | 53.0 (16.5) | −6.4 (−10.9, −1.8) | 0.003 |
| Extremely | 82.0 (13.0) | 18.0 (2.8) | 49.0 (15.5) | 35.0 (10.9) | −7.2 (−10.8, −3.7) | <0.001 |
| CI = confidence interval., WOCF = worst observation carried forward. Composite estimand: Data considered non−response by using WOCF (including the baseline value) after initiation of rescue treatment or after permanent discontinuation of IMP. Missing data imputed using WOCF (including the baseline value). Cochran−Mantel−Haenszel test, stratified by study ID, region, baseline IGA−CHE and baseline HEIS dimension response. | | | | | | |

**Table S3.** Mean time in response from baseline to week 16 for clinically significant improvements in DLQI, HEIS, HEIS PDAL and HEIS Emb with delgocitinib cream or cream vehicle

|  | **Delgocitinib cream 20 mg/g** | **Cream vehicle** | **Difference  (95% CI)** | **p-value** |
| --- | --- | --- | --- | --- |
| **DLQI ≥4-point improvement** **from baseline to week 16** | | | | |
| Patients with baseline score, n | 604 | 301 |  | |
| AUC (SE) | 80.5 (1.5) | 53.2 (2.3) | 27.2 (21.8, 32.6) | <0.001 |
| **HEIS ≥1.3-point improvement** **from baseline to week 16** | | | | |
| Patients with baseline score, n | 583 | 288 |  | |
| AUC (SE) | 61.9 (1.7) | 33.4 (2.3) | 28.5 (22.9, 34.0) | <0.001 |
| **HEIS PDAL ≥1.3-point improvement** **from baseline to week 16** | | | | |
| Patients with baseline score, n | 581 | 288 |  | |
| AUC (SE) | 66.5 (1.6) | 41.8 (2.4) | 24.6 (19.0, 30.3) | <0.001 |
| **HEIS Emb ≥1.5-point improvement** **from baseline to week 16** | | | | |
| Patients with baseline score, n | 580 | 288 |  | |
| AUC (SE) | 64.2 (1.7) | (35.8 (2.3) | 28.4 (22.8, 34.1) | <0.001 |
| Analysis of covariance, adjusted for study ID and baseline value of variable: estimated treatment coefficient and 95% CI. Primary analysis of the composite estimand strategy with missing data imputed as non-response for both groups. Data after initiation of rescue treatments or permanent discontinuation of study treatment are treated as missing. Two-sided p-values are reported.  AUC, area under the curve; CI, confidence interval; n, number of patients; SE, standard error. | | | | |

**Figure S1. Estimated cumulative incidence of patients achieving a minimum clinically important improvement in (A) DLQI, (B) HEIS (C) HEIS PDAL and (D) HEIS Emb over 16 weeks of treatment with delgocitinib cream or cream vehicle.**

**
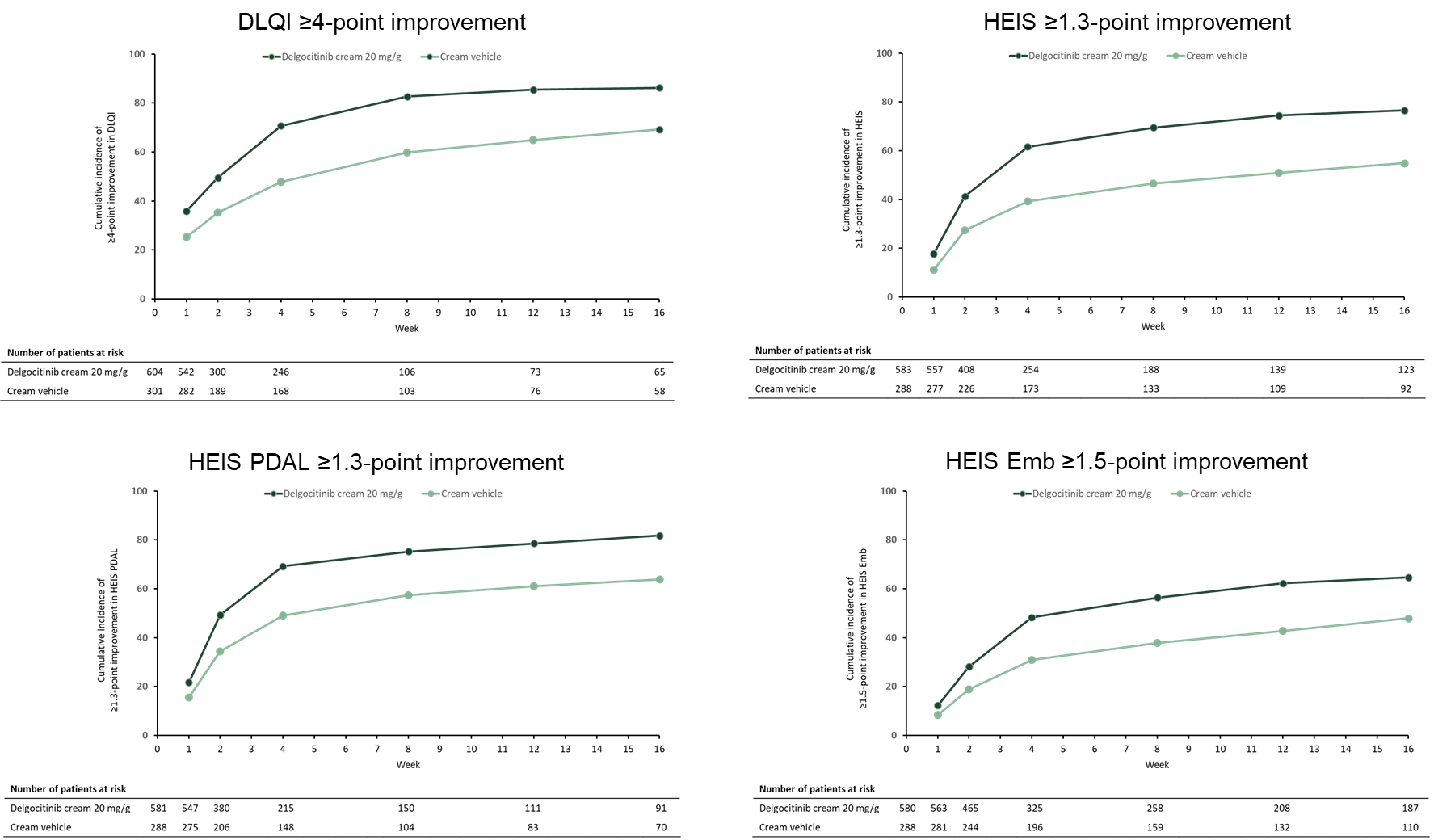
**
